# Topics of Public Interest

**Published:** 1907-03

**Authors:** 


### TOPICS OF PUBLIC INTEREST.

### An Abstract of Various Views in Medical Licensure.

By E. S. McKEE, M. D., Cincinnati.

*(St. Louis Medical Review, February 6, 1907.)*

RECIPROCITY was given a good deal of consideration by the President of the American Medical Association, at Boston, in his presidential address. Dr. Mayo thinks that what is needed is a higher standard of requirements and more and better supervision of professional schools. At the present time each State has its own standard of requirements. The conditions now with reference to reciprocity in medical license are well-nigh intolerable, and restrain the individual freedom guaranteed by the Constitution. The borders between the states are imaginary lines, yet a physician on one side of the border can not relieve human suffering on the other side without becoming amenable to the law or subjecting himself to vexatious examinations which he has already successfully passed in his own state. This must be met, and speedily, by agreement between examining boards as to the minimum of requirements. After all, this is but a part of the educational problem. If we can solve this, licensing boards could at once adopt more uniform examinations and reciprocity.

UNRESTRICTED RECIPROCITY IMPOSSIBLE.

Dr. James A. Egan, secretary of the Illinois State Board of Health, has, in a paper before the Illinois State Medical Association, shown this to be true as to his view. The Illinois State law, for example, distinctly states that a license to practise medicine shall be given only after satisfactory examination, and in making provision for reciprocity it distinctly states that this shall be allowed only in the case of certificates issued by other boards after examination. The old practitioner, the one who is not so very old in fact, but in practice before a medical examination was required in this state, and who was consequently licensed without an examination, is barred from practice in Illinois without an examination, either in his own state, if that has reciprocity with Illinois, or in Illinois. Illinois has, however, gone farther than any other state to mitigate the laws in this respect. It allows a credit of 5 per cent. on the required 75 per cent. of successful answers for each five years the candidate has been in active practice. Certainly a very fair and considerate arrangement. Illinois has gone the limit in reducing this hardship to a minimum. If all the other States would follow the example of Illinois they would be as wise as just. If the practice of medicine can not be taken out of the State and placed in the interstate laws, the hardship resulting in individual cases could be materially mitigated by the adoption by the various states of some such regulation as, according to Dr. Egan, has been adopted by his State.

RECIPROCITY A QUESTION OF STATES' RIGHTS.

The Editor of the *Medical Record* (July 28, 1906), discusses the remarks of President Mayo at Boston in his desire that an agreement may be reached whereby a legalised practitioner in one state may be recognised as such in every other state. Theoretically, the editor thinks this is a very desirable state of affairs, but practically, at least till the older generation of physicians shall have passed away, an impossibility. He agrees that the conditions are "well-nigh intolerable" but does not assent to the "boundaries between states being imaginary lines." The doctrine of states' rights was shorn of some of its exaggeration in the '60s, but it exists, and must so long as the present constitution of the United States continues in force. The editor does not wish to be put down as opposed to reciprocity in medical licensure, but is, on the contrary, in favor of the fullest reciprocity compatible with the laws of the individual states.

TEN YEARS' PRACTICE EQUIVALENT TO A LICENSE.

Dr. W. L. Rodman, of Philadelphia, makes the excellent suggestion that ten years of practice should be considered the equivalent of passage of an examination for license to practise. Any physician holding the diploma of a reputable college, who has had ten years' experience in practice, certainly should be permitted to practise anywhere in the United States.

A UNIFORM EDUCATIONAL BASIS THE SOLUTION.

Dr. William Warren Potter, of Buffalo, editor of the *Buffalo Medical Journal,* wrote for that most excellent series of prize essays in the *New York Medical Journal* (January 20, 1906) on the subject of Reciprocity. His essay very justly took the prize. He thinks that when the preliminary or entrance requirements, the length of terms and methods of a collegiate training, and licensing examinations are placed upon a uniform basis throughout the country, the problem of reciprocity in medical licensure will be solved. This he thinks to be the only equitable solution of the perplexing question. "Of what avail is reciprocity unless founded on justice? Surely an enforced interchange of licensing courtesies would be worse than none; it would be irritating, unfair and ephemeral. Whereas, if founded upon uniformity of standards, it would be pleasing, just and enduring. It seems strange, almost absurd, that entrance requirements have not already been placed upon a basis of uniformity. It is well-nigh scandalous that it has not been established. Ignorance should not be tolerated anywhere among those who contemplate entering upon the study of medicine and the colleges throughout the length and breadth of the land should agree without unnecessary delay upon the minimum of preliminary or entrance qualifications. This once done it would be a comparatively short step to the standardisation of the methods of teaching, as well as the length of the undergraduate terms. Finally, with these two essentials agreed upon, the licensing boards would be compelled to establish a uniformity of methods of examinations, and, likewise, the states could no longer withhold indorsements of licenses.

"The Association of American Medical Colleges can be depended upon to establish the first two propositions, namely, the uniformity of entrance requirements and the length of collegiate terms and methods of teaching. It will then be 'up to' the examining boards and State authorities, to use a trite expression of the day, to see that their methods become of uniform standard and that reciprocity is established with promptitude.

"I am persuaded, from considerable observation and experience, that interstate reciprocity in licensing is best accomplished through the avenues named, and, indeed, that only in this way can it be accomplished with fairness to all the variant interests, with lasting credit to the profession, and with substantial benefit to the licensees who expect to derive advantages from this privilege."

RECIPROCITY AND INDIVIDUALISM.

From the young and vigorous editors of the *Buffalo Medical Journal* and *New York Medical Record,* and the secretary of the Illinois State Board of Health, the "pattern" saint of licensing bodies, we will now turn to Dr. Deering J. Roberts, the long-time editor of the *Southern Practitioner* of Nashville, who acknowledges his age, and that a quarter of a century ago in his address before the general session of the American Medical Association as chairman of the section on State Medicine he was more or less active in opposing the efforts at that time, instigated as he then thought and still thinks by misguided members of our profession, to establish a regulation of the medical profession by an appeal to the various state legislatures. Dr. Roberts may be wrong in some respects, but as late surgeon C. S. A. and a practitioner and teacher for forty-five years he has probably done more to alleviate human misery directly or indirectly than any two of the others mentioned. The doctor reviews the history of medical legislation for some fifty years, and fails to see that the advance which has been made in medicine is due to the regulation procedures of the various legislatures. He grants that medicine and surgery have made great strides, and he hopes they will continue, but, as in former years, it will be due to the individual and combined efforts of the profession equally as well without as with the aid of the law.

Reciprocal relations are such that Dr. Roberts thinks it will be many a day before we can get the different states to agree upon any definite and uniform plan. From personal observations he can cite that the same board has passed undergraduates and rejected graduates of the same school and even in some instances has passed those who have failed to secure the degree after the full four years' course and an examination by the faculty of the school.

The *Southern Practitioner's* editor suggests that the retired medical officers of the Army, Navy and the Marine-Hospital Service be authorized to make examinations of all who may apply, a certificate of approval to qualify them to practise medicine and surgery in the District of Columbia and all the Territories. Said certificate, he confidently believes would be recognised by nearly if not all of the states. Boards of three members each could be readily convened annually in the different sections of the Union, at some central, convenient city, to which recent graduates and older members of the profession could apply for reexamination if they desired to have free range in the days to come in which to pursue their vocation.

These retired medical officers are all well qualified and competent, they have no very strong local or political affiliations, their loyalty being more to the national government as a whole and the United States at large, rather than to any special local environment. The fees would add something to their reduced pay, as having been placed on the retired list of their special branch of service, and the duties would not be onerous to them.

RECIPROCITY IN MEDICAL LICENSURE.

Dr. Albert Vander Veer, of Albany, Regent of the University of the State of New York, states in the *New York Medical Journal* for October 13, 1906, that New York, New Jersey, Michigan and Ohio have entered into a reciprocity agreement based on examination only in any one of them. The preliminary education required for admission to the medical schools must be the same in each state. The unit of value in measuring or estimating the preliminary qualifications is the count which is of universal use in New York State, and is in accord with the academic syllabus for the secondary schools. The following interesting facts are related:

"From the report of an American consul in Austria, Hugo Donzelmann, Prague, March, 1898, the following statement of the principles of the first laws promulgated in Europe with reference to the practice of medicine and pharmacy is condensed. Their principle is still apparent in all later laws, namely, the public good, and this principle is the underlying principle in the growth of laws affecting admission to the practice of the learned professions in the United States.

"The first decree of Frederick II., in 1224, can be said to have been the fundamental constitution of all existing laws in Europe, the same having been amended and improved upon from time to time, but always bearing in mind that the practice of medicine and pharmacy was to be under the special care and supervision of the government in order to protect its people against imposition. In that year he established by a decree the first college for the education of physicians, at Naples, and promulgated the first laws governing the practice of medicine within his domain, viz., that no person should be admitted to the practice of medicine who had not passed his examination before the Collegio Medico di Napoli; that after having received his diploma from said college it became necessary for the person to enter into active practice with a regular practising physician for the period of one year as assistant; and that an oath had to be taken by the person whereby he promised to follow and live up to the laws of the country respecting the practice and sale of medicine and whereby he bound himself to attend to the sick, to accept only a reasonable fee from all who were able to pay, and to treat the poor and impecunious free and without charge."

The New York statute establishing these standards whereon reciprocity may be entered into with other States, reads as follows:

"Applicants examined and licensed by other State examining boards registered by the regents as maintaining standards not lower than those provided by this article, may, without further examination, on payment of $10 to the regents, and on submitting such evidence as they may require, receive from them the endorsement of their license or diploma, conferring all rights and privileges of a regents' license issued after examination."

In determining whether standards are lower than those provided by the statute, four distinct lines of statutory requirements are studied: (1) the preliminary education required for admission to the professional school; (2) the professional training required for graduation; (3) the licensing test required by an independent examining board: and (4) registry in offices of record.

The present preliminary education of New York State required for admission to registered medical schools and the medical licensing examination, is evidence of four full years of secondary education subsequent to eight years of elementary or the equivalent in higher institutions. The evidence of this preliminary education is the medical student certificate, in the determination of which certain specific requirements are exacted.

The professional requirement for the registration of medical schools and for admission to the medical licensing examination is the study of medicine not less than four full school years or at least nine months each, including four satisfactory courses of at least six months each in four different calendar years in a medical school registered as maintaining at the time a satisfactory standard.

The formal licensing examination is but one of the three essentials to a license to practise medicine in the State. The first essential is based on the principle that the student had completed his preparatory work prior to entrance on his professional, with the possibility of his being conditioned in not more than one year of the preliminary training, *which must be worked off before beginning the second course counted toward the degree.*

*The second essential,* a degree from a registered professional school, is a marked step in advance of the requirements in foreign countries, as it is well known that very few of the medical practitioners of Germany and Russia complete the requirements for a degree, the State's examination being regarded as even a higher qualification.

*The third essential* is the formal written licensing examination, which is based on the former essentials and is the final act in the government's special care for the public good. The question of the use of English does not enter into the revision of the applicant's paper, although he is obliged to use the English language in his answers, for his mental training was determined by the first essential requirement. No effort is made by the formal written test to determine the clinical experience of an applicant, such requirement having been met under the second essential. The final test—the licensing examination—determines the competency of the candidate applying for admission to the *general* practice of medicine in the State, in order to protect the people against imposition.

The importance of these agreements can hardly be overestimated, and to show the marked advance of this step Vander Veer quotes from Bulletin 8, *Professional Education in the United States,* entitled, *Medicine,* issued January, 1900:

"A uniform standard for admission throughout the United States is impracticable at present, owing to varying conditions as to density of population, educational advantages, and general development. Weak States cannot maintain the standards maintained elsewhere, and strong States cannot afford to lower their standards. The present need of less multiplication of standards, however, is most important. Instead of a separate standard for almost each political division, two or at most three standards should answer for all. In the first group should come the strongest States, and the standards maintained by these States would act as a stimulus to weaker political divisions."

From a careful consideration of the *synopsis* published in *Handbook* 9 of the education department, it plainly appears that reciprocity can become effective in the near future between a group of States extending from the Atlantic to the Pacific.

THE HARDSHIPS OF EXISTING CONDITIONS.

Pathetic correspondence is contained in recent numbers of the *American Journal of Clinical Medicine* from two elderly doctors who were compelled to move from the East to Oregon and and Washington, the one for the benefit of his wife's health, the other for the good of his own health. For nearly thirty years, one of them writes, he had been a regular practitioner, a graduate, a registrate, a member of a society recognised by the A. M. A., and yet he is not fit to practise in Oregon. He says: "Here I am, a man fifty-four years of age, too old to be very successful in any other line of business, an invalid wife, no means to speak of, and yet I shall not have the means to earn my bread and butter. All this in a profession in which I have had so much pride, which I regarded as the noblest and kindest, but which, on the Coast, seems to desire to Oslerize old Eastern brethren. There is need of a little justice, fair play and the use of the Golden Rule. Medical legislation is not to keep one honest man from competing with another, but to keep quacks, charlatans and thieves from preying upon the public. It is foolish to say that a man fit to practise in one State is not in another."

HOW MAY INTERSTATE RECIPROCITY BE BEST ACCOMPLISHED.

Do away with ignorance among those entering the profession of medicine. The minimum of preliminary or entrance qualifications should be agreed upon without delay. Follow the example of Illinois and mitigate the laws as much as possible for the old practitioner by allowing him a rebate, as it were, of five per cent. for every five years he has practised. An examination or degree should be given by the Federal Government, not unlike that recommended by Dr. Roberts, which license would be received with honor by most if not all the States. Much wrong is evidently done men who have practised for twenty-five, thirty, or more years and are compelled to remove to another State. Selfishness is at the bottom of much of this discrimination, and should give way to broader and more generous views. It is un-American.

---

### Medical Society of the State of New York.

### Action of the House of Delegates Relating to the Medical Society of the County of Erie.

[*From New York State Journal of Medicine, February, 1907*]

IN view of the fact that the Medical Society of the County of Erie, through its attorney, is of the opinion that certain portions of the constitution and by-laws of the Medical Society of the State of New York, and of the advisory by-laws sent out for adoption to county societies, are in conflict with the statute laws of the State of New York, the Ad Interim House of Delegates, at a meeting held in New York, December 8, 1906, resolved to consult the Hon. William G. Choate, former Justice of the United States Court, with the idea of ascertaining his views on the subject and of preparing a bill for the purpose of remedying any defects that may exist. The opinion of Judge Choate, and the bill, are herewith appended, and it is proper to add in this connection, that the views held by this eminent jurist are also held by the counsel of the Medical Society of the State of New York, Mr. James Taylor Lewis.

OPINION OF MR. WILLIAM G. CHOATE.

I have carefully examined all the laws of this State relating to the Medical Society of the State of New York and to the State Medical Association; also the entire proceedings for the consolidation of the two societies, for the purpose of advising you as to my views with reference to the introduction of a bill at the coming session of the Legislature. I have reached the following conclusions:

First: Although by the early statutes certain persons were made permanent members or delegates, and although certain institutions were permitted to send delegates to the annual meeting of the Medical Society of the State of New York, there is no doubt whatever that the legislature has the power to change or modify the laws of 1806 or 1813, and prescribe different qualifications and methods of election of members. By section 23 of the laws of 1813, that act was expressly made, subject to modification, amendment or repeal, and was declared a public act.

Secondly: Before consolidation of the two societies, chapter 549 of the laws of 1904, was enacted, in effect amending section 3 of the laws of 1813. By its terms full authority was given to your society to elect such members as may be provided for in the constitution and by-laws of the society; "said Medical Society being empowered to fix and determine the qualifications and conditions of membership therein, and to regulate and control its own membership." Chapter 544 of the laws of that same year gave the society the privilege of adopting a constitution and by-laws relative to the admission and expulsion of members and the regulation of its affairs, and sections 5 and 7 of the laws of 1813 were expressly repealed. The act authorizing consolidation, chapter 1 of the laws of 1904, provided that the consolidated corporation should have all the powers, rights and privileges possessed by either corporation, at or immediately prior to the consolidation. The order consolidating the two corporations was entered after all these laws were passed. The consolidation agreement and the order, adopted a constitution and by-laws by which the same full power with regard to membership was vested in the consolidated body. I am of the opinion, therefore, that you need no further legislation in any manner referring to your membership.

Thirdly: By the same course of reasoning, I believe the establishment of the House of Delegates, the Council and Censors are now complete and in accord with legal enactments.

Fourthly: The society has ample power to collect its dues. The special charter granted the association at section 5 thereof, gave that corporation the power to determine the amount of the annual dues and also to impose assessments and to collect the same by suit or otherwise. The consolidation, therefore, brought to the present state society this provision, which in effect removed the limitation contained in chapter 682 of the laws of 1893, which act permitted but five dollars to be imposed as dues and assessments in any one year. To the same effect were the provisions of chapter 549 of the laws of 1904, which gave to the society the power to fix and determine the conditions and qualifications of membership therein, and to regulate and control its own membership.

Fifthly: There is but one matter upon which legislation is desirable. I am of the opinion that a broad power given to the state society to determine what county societies are in affiliation with it, and the conditions on which such affiliation shall continue, will give the society all the power it needs.

Sixthly: Under the present statutes the state society may hold $150,000 in property; if you think it advisable to enlarge that amount, it may be accomplished by legislative enactment.

Seventhly: There may possibly be doubt as to whether the property of the society is exempt from taxation. In 1903, county medical societies were apparently considered as not coming within exemptions of scientific societies. If it is desirable to make this exemption certain, a separate act should be passed amending subdivision 18 of section 4 of the tax law.

I am therefore of the opinion that the Medical Society of the State of New York, as now constituted under its by-laws and constitution, can go into operation and continue business without embarrassment, even without any further legislation, and the acts of the **ad interim** House of Delegates performed under the authority of the order of consolidation and the constitution and by-laws of the society are entirely legal so far as they conform to the authorisations contained therein.

The principal point seems to be that there may be rights of permanent membership heretofore created by statute inconsistent with the absolute right of the society to regulate its membership. There is no objection to adding to your act a section providing that all laws and parts of laws creating permanent members or delegates to the Medical Society of the State of New York inconsistent with chapters 1, 544 and 549 of the Laws of 1904, or with the order of court consolidating the two corporations made in pursuance thereof, are hereby repealed.

If you consider that some members would be satisfied only with a legislative re-enactment of powers which the state society now has, I know of no legal reason why that should not be done, but such a course I consider absolutely unnecessary, and my advice is, therefore, opposed to such action.

AN ACT IN RELATION TO THE MEDICAL SOCIETY OF THE STATE OF NEW YORK.

The People of the State of New York represented in Senate and Assembly do enact as follows:—

Section 1. The Medical Society of the State of New York, reconstituted and continued by the consolidation of the Medical Society of the State of New York and The New York State Medical Association, in accordance with the terms of chapter One of the Laws of Nineteen Hundred Four, shall have power from time to time to determine what county medical societies are in affiliation with it, and to prescribe the terms and conditions under which any county medical society shall be or shall continue to be so affiliated, and shall have power to suspend and discipline affiliated county medical societies.

Section 2. All acts or parts of acts, relating to permanent members or delegates in, or to delegates to the Medical Society of the State of New York, inconsistent with the provisions of Chapters Five Hundred Forty-four and Five Hundred Forty-nine of the Laws of Nineteen Hundred Four, or inconsistent with the order of the Supreme Court made and entered December Ninth, Nineteen Hundred Five, pursuant to the provisions of Chapter One of the Laws of Nineteen Hundred Four, so far as they provide for such members or delegates, are hereby repealed.

Section 3. This Act shall take effect immediately.

---

### The Prevention of Unnecessary Blindness.

THE NEW YORK STATE COMMISSION to Investigate the Condition of the Blind has issued the following circular which should receive the careful attention of every medical man in active practice. It is important that the commission should be in possession of all the facts possible to obtain on the points mentioned at an early day.

*Editor Buffalo Medical Journal*: SIR: The loss of sight in the case of an individual is of economic importance to the state. The New York State Commission to Investigate the Condition of the Blind has been charged, therefore, by the legislature with the duty of inquiring into the causes of blindness, and of recommending methods by which, as far as possible, unnecessary blindness may be prevented. To that end the commission begs the assistance and advice of the medical profession. The secretary will gratefully receive and acknowledge any reprints, reports, pamphlets—or personal communications bearing on the causes and prevention of blindness.

More especially, information is sought on the following points:

*Congenital Blindness*:

Its causes, the influence of heredity, consanguinity, etc.

The reports of cases of blind parents producing blind children, character of blindness in such cases, and any other material facts relating to this phase of the topic.

*Ophthalmia Neonatorum*:

How generally are preventive measures employed?

Statistics bearing on the subject.

What silver salt and in what strength should be recommended?

*Trachoma and Other Infectious Eye Diseases*:

Statistics.

How may early treatment be secured?

Prevalence in schools, orphan asylums, etc.

Preventive measures—medical inspection of schools.

*Blindness From Accident, Injuries, Fireworks, Toy-pistols, Etc.*:

Statistics.

Method of protection for eyes of workmen, and others.

Prohibition of dangerous explosives at celebrations.

*Toxic Amblyopia*:

From methyl alcohol—other toxic agents. How may the public be protected?

*Neglect on the Part of Patients Visiting Dispensaries*:

Patients suffering from conditions threatening vision absent themselves from clinics after being advised of the need of immediate treatment until too late.

Method of reaching such.

*Blindness Due to Neglect of Slight Ophthalmic Injuries*:

How can early treatment be more generally secured?

*Blindness Due to Improper Hygiene and Sanitation in Corneal Troubles of Children*:

How can early treatment be secured?

*Other Causes of Blindness*:

Suggestions as to prevention.

The Commission will be most grateful for advice and assistance on the above subjects.

F. PARK LEWIS, *President*,
454 Franklin St., Buffalo,
O. H. BURRITT, *Secretary*,
Batavia.

Batavia, N. Y., December 15, 1906.

---

### The Prevention of Unnecessary Blindness.

THE COMMITTEE ON OPHTHALMIA NEONATORUM, of the American Medical Association, has issued the following:

*To the Members of the American Medical Association*:

While much has been written and many laws have been passed having as their object the protection of the eyes of newborn children, an appalling amount of blindness results each year from the neglect of preventive or curative measures in ophthalmia neonatorum. It is a serious criticism upon our efficiency and our position as a scientific body that a disease the prevention and effective treatment of which are so well understood should be allowed to cause such widespread disaster. It would seem that the time has come when ophthalmia neonatorum should be completely and permanently stamped out as a cause of infantile blindness. This can be accomplished, however, only by the concurrent action of the whole medical profession throughout the country.

In recognition of this fact the following resolutions were adopted at the last meeting of the American Medical Association:

**Whereas,** Notwithstanding the long-continued efforts of the medical profession to make generally known the infectious character of ophthalmia neonatorum and its danger to sight, the ranks of the blind are still largely increased annually by those who have unnecessarily lost their vision as a result of this disease; and

**Whereas,** We possess in the silver salts an almost absolute specific for its prevention and treatment, therefore, be it

**Resolved,** That this Section recommends that a committee consisting of at least one ophthalmologist, one obstetrician, and one sanitarian, with invited co-operation of a sub-committee, consisting of the president and secretary of each state society, be appointed by the President of the Association to formulate and make effective the details of a plan that may give uniform legislation and definite instruction to the profession and laity concerning the prevention and treatment of this disease.

**Resolved,** That this Section recommend an ophthalmologist for such committee to be appointed by the incoming Chairman and Executive Committee.

In fulfilling the directions of the association, the committee desires to present the following program, and it is earnestly requested that expressions of approval or of criticism be freely submitted for its guidance so that the recommendations of the committee shall be in effect those of the entire profession.

While it is true that merely washing out the lids of the new-born child with distilled water lessens largely the number of cases of ophthalmia neonatorum, the difficulty of this procedure in inexpert hands, and the probability of cocci remaining in the eyes of the child, make essential the employment of some germicide; and by almost universal agreement one of the salts of silver is recognised as the most nearly specific.

1. As experienced pharmacists give the assurance that a solution of nitrate of silver, when hermetically sealed in light-proof receptacles will keep indefinitely, and as physicians are generally agreed that this is the most effective silver salt for the prevention of ophthalmia neonatorium as well as the most inexpensive, the choice of the committee would seem to be limited to this as the special germicide to be recommended.

Does this meet with your approval?

2. As the classical 2 per cent. of Crede occasionally causes silver catarrh and 1 per cent. rarely does so, and since as a preventive measure it must be used largely by students, midwives and other inexperienced persons, would it not be safer and better to select the 1 per cent. solution as the strength of nitrate of silver to be recommended?

In this connection the following statements from an address delivered by R. Brudenell Carter, at London, in 1902, are pertinent:

"There can be no possible reason why the instructed midwives whom we are hoping to obtain in the near future should not universally adopt this (Crede's) preventive treatment, and thus place the children of their patients beyond the reach of danger. The application ordered by Prof. Crede appears to me to be unnecessarily strong. For curative purposes I have always found a solution of one part in 240, or of two grains instead of ten to the fluid ounce of water, to be perfectly satisfactory; and I have no doubt that it would be equally so as a prophylactic."

Do you recommend a 1 per cent. solution of nitrate of silver as a prophylactic in all cases?

3. As the failure to use the silver salt as a routine part of every new-born child's toilet is largely due to the fact that the preparation is not at hand when required, should not this be met by the enactment of a law in each state securing the distribution through the various state health boards of small, sealed, inexpensive, light-proof, tubes or ampoules of the selected solution to be sent gratuitously to every physician and midwife on the passage of the law and subsequently when required or on the filing of each birth certificate?

Do you approve of this?

4. In order that this solution, which shall be freely supplied, may be always used, it shall be required that the birth certificate shall contain a statement signed by the accoucheur that some recognised procedure specified on the blank has been used in each eye of the new-born child on the day of birth as a preventive of ophthalmia neonatorum; in the absence of this statement should ophthalmia neonatorum develop and the child lose the sight of one or both eyes, the accoucheur, whether physician or midwife, filing the certificate, shall be deemed guilty of criminal negligence and shall be subject to such penalty as the state may exact.

Does this meet with your approval?

5. It shall be the duty of the State Health Officer to place ophthalmia neonatorum on the list of communicable diseases, and failure of the attendant to report to the local health board each birth, the existence of ophhalmia neonatorum when present, and the fact that some prophylactic measure has or has not been used, shall be considered a misdemeanor and subject the offender to such penalty as the state may exact.

Do you approve of this?

6. It is strongly urged if the above program meets with general approval that the president of each state association be invited to appoint a Committee on Ophthalmia Neonatorum whose duty it will be to collaborate with the State Board of Health in securing in the several states the enactment and enforcement of laws embodying the above propositions.

Do you approve of this?

The committee will feel grateful for a categorical reply to each of these questions and such further suggestions or comments as you may wish to make concerning this important subject.

F. Park Lewis, *Chairman*, Buffalo, N. Y.
J. Clifton Edgar, New York, N. Y.
F. F. Wesbrook, Minneapolis, Minn.

---

